# Editorial: Understanding the Importance of Non-Canonical tRNA Function

**DOI:** 10.3389/fmolb.2021.769784

**Published:** 2021-10-07

**Authors:** Juan Pablo Tosar, Pavel Ivanov, Lluís Ribas de Pouplana, Adrian Gabriel Torres

**Affiliations:** ^1^ Functional Genomics Unit, Institute Pasteur de Montevideo, Montevideo, Uruguay; ^2^ Faculty of Science, Universidad de la República, Montevideo, Uruguay; ^3^ Division of Rheumatology, Immunology and Allergy, Brigham and Women’s Hospital, Boston, MA, United States; ^4^ Department of Medicine, Harvard Medical School, Boston, MA, United States; ^5^ The Broad Institute of Harvard and M.I.T., Cambridge, MA, United States; ^6^ Catalan Institution for Research and Advanced Studies, Barcelona, Spain; ^7^ Institute for Research in Biomedicine, The Barcelona Institute of Science and Technology, Barcelona, Spain

**Keywords:** transfer RNA, tRNA-derived fragments, tRNA modifications, tRNA processing, non-canonical function, gene regulation, human diseases, evolution

The last universal common ancestor of all current forms of life already contained transfer RNAs (tRNAs) serving as adaptor molecules between nucleic acids and proteins. However, only in recent years has it become clear that the functions of these ancient RNA molecules expand beyond their central role in protein synthesis by the ribosome (Schimmel, 2018). The goal of this Research Topic is to revise our current knowledge on these non-canonical tRNA functions and create a forum for discussion that would expand and stimulate this field of research. Here, we are delighted to present a Research Topic of articles that address the importance of non-canonical tRNA functions from molecular, biochemical, evolutionary, and biomedical perspectives.

In the review by Avcilar-Kucukgoze and Kashina, key non-canonical tRNA functions in eukaryotes are introduced and elegantly presented. The focus is placed on the involvement of tRNAs in diverse cellular processes such as stress response triggered by amino acid starvation, regulation of mitochondria-triggered apoptosis, protein arginylation and other tRNA-mediated post-translational modifications, priming of retrotransposons, adaptive mistranslation by tRNA mischarging, and regulation of biological processes by tRNA-derived fragments (tRFs) including tRNA-derived stress-induced RNAs (tiRNAs) and tRNA halves (Ivanov et al., 2011).

Extracellular RNAs (ex-RNAs) are involved in cell-to-cell communication and may act as a potential source of biomarkers for human diseases, but the roles of ex-tRNAs or their fragments (ex-tRFs) in these processes have not been widely studied, despite being among the most abundant ex-RNAs (Tosar et al., 2020). Torres and Martí discuss the biology of extracellular tRNAs and tRFs and propose a critical role for post-transcriptional tRNA modifications in ex-tRNA/ex-tRF recognition, stability, uptake and function in recipient cells.

MicroRNAs (miRNAs) are small non-coding RNAs that associate with argonaute (Ago) proteins and repress translation upon binding to target messenger RNAs (mRNAs). MiRNA/mRNA interactions are based on binding of the 5′-end of the miRNA to the 3′-end region of mRNAs (herein, 5′ miRNA-mRNA forward orientation). Previous reports have shown that certain tRFs can perform miRNA-like Ago-mediated mRNA silencing (Kumar et al., 2014; Kuscu et al., 2018; Guan et al., 2020). However, tRF/mRNA recognition rules are still not completely understood. Guan et al. analyzed CLASH (crosslinking, ligation, and sequencing of hybrids) and Ago PAR-CLIP (photoactivatable ribonucleoside-enhanced crosslinking and immunoprecipitation) datasets, in order to identify potential motifs dictating tRF/mRNA interactions. The novelty of their work rely on considering not only forward (5′ tRF-mRNA), but also reverse (5′ mRNA-tRF) oriented CLASH chimeras. By doing so, the authors improved the discovery of sequence motifs potentially involved in tRF/mRNA interaction, found a clear asymmetry of the paired tRF and their targets in both orientations suggesting alternative modes of tRF/mRNA recognition, and provided a comprehensive list of candidate tRFs with target-binding motifs worth for future experimental validation.

Analyses of tRNA gene (tDNA) composition reveal a high redundancy at the organismal level that cannot be fully explained by the need to express cognate tRNAs (Torres, 2019) (see also below). Furthermore, some organisms (i.e., Archaea) even reconstitute their tRNAs from split tDNAs. These split genes code for different tRNA parts that can assemble into the full-length mature tRNA post-transcriptionally, and could possibly be an ancestral trait rather than a group-specific genomic innovation (Kanai, 2015). In an opinion article, Grigoriev explores the possibility that tRFs could be precursors to modern RNA interference (RNAi) mechanisms based on analyses of tDNA patterns. Transcripts deriving from tDNA-like genes and split tDNAs could have been a source for small non-coding RNAs (i.e., tRFs) that could have been loaded into primordial Ago proteins and exert early RNAi-like functions. This would support the function of tRFs being ancestral, possibly preceding the hypothetical genomic condensation event of split tRNA genes into modern tDNAs. Because the sequence of tRFs is constrained by their parental tRNA structure, new hairpins could have evolved from non-coding genomic regions later in evolution, thus providing a new potential source of small RNAs with higher sequence flexibility (i.e., extant small regulatory RNAs). Nevertheless, tRFs are still involved in fundamental processes to date, so they would have likely conserved part of their original functions.

Analyses of tDNA variants can also give further insights into potential non-canonical tRNA functions. The work by Ehrlich et al. studies tDNA composition in the three domains of life and focuses on those clades that are missing specific tRNA genes. Some tDNAs are lost to prevent decoding ambiguities. For example tDNAs encoding tRNAs with A or G at the first position of the anticodon tend to be mutually exclusive (Grosjean et al., 2010; Novoa et al., 2012). However, exceptions to such anticodon-sparing strategies exist in several species (Maraia and Arimbasseri, 2017), questioning the strength of the selection forces that shaped tDNA-loss patterns. By using updated tRNA gene databases coupled to manual curation of predicted tDNAs, the authors found that many of these exceptions were tDNAs that were unlikely to produce *bona fide* tRNAs. Although much of the evolutionary pressure shaping tRNA gene features is probably linked to translation fidelity (i.e., canonical tRNA function), the authors made a case that tDNAs that can potentially produce *a priori* non-*bona fide* tRNAs may be a source for tRNA variants with non-canonical functions.

More comprehensive and reliable methods for tRNA/tRF detection and quantification are being rapidly developed. These now also include the possibility of detecting and mapping post-transcriptional RNA modifications at single-nucleotide resolution, and evaluating tRNA processing steps. Thus, we are looking forward to new technical and conceptual developments that will surely push the field beyond its current status. Meanwhile, we invite the readers to dive into this Research Topic of papers to get a glimpse of the function of tRNAs beyond translation. Even if these functions represent just the tip of the iceberg of what is still left to be uncovered.
